# Assessing the Quality of YouTube Videos About Cannabinoid Hyperemesis Syndrome

**DOI:** 10.7759/cureus.109113

**Published:** 2026-05-18

**Authors:** Larissa Dean, Michelle A Padley, Hunter T Pham, Mary Finedore, Annie Vu, Jillian A Sargent, Christine Skovira, Christian Kolacki, Lindsey Ouellette, Bryan S Judge, Brad D Riley, Jeffery S Jones

**Affiliations:** 1 Medicine, Michigan State University College of Human Medicine, Grand Rapids, USA; 2 Emergency Medicine, Michigan State University College of Human Medicine, Grand Rapids, USA; 3 Emergency Medicine, Corewell Health, Grand Rapids, USA; 4 Medicine, Michigan State University College of Human Medicine, East Lansing, USA; 5 Trauma and Acute Care Surgery, Michigan State University College of Human Medicine, Flint, USA; 6 Emergency Department, Spectrum Health Medical Group, Grand Rapids, USA

**Keywords:** cannabinoid hyperemesis syndrome, cannabis use, effects of social media, mixed methods research, qualitative content analysis

## Abstract

Objective

This study aimed to evaluate the accuracy, completeness, and clinical usefulness of YouTube (Alphabet Inc., Mountain View, CA) videos about cannabinoid hyperemesis syndrome (CHS).

Methods

This cross-sectional study assessed YouTube videos that were identified using standardized CHS-related search terms and predefined eligibility criteria. Trained reviewers used an author-developed CHS content checklist and a global usefulness scale for each video, with any discrepant or complex cases resolved by medical toxicologists. Outcomes included accuracy across key CHS domains, overall clinical usefulness, and the association between video quality and basic engagement metrics.

Results

A total of 97 videos were analyzed, with a mean length of 9.8 minutes and a cumulative view count of 795,264. Only 25.8% of videos were rated as useful and 2.1% as exemplary, whereas 52.6% were rated not useful and 19.6% as misleading due to missing essential content and/or unsubstantiated claims. Personal testimonial videos were common and often combined accurate symptom descriptions with speculative etiologies and non-evidence-based management advice. Engagement metrics showed little meaningful association with reviewer-rated accuracy or usefulness, and several of the most-viewed videos contained substantial misinformation. Interrater agreement for key classifications was substantial (κ = 0.78).

Conclusions

CHS-related information on YouTube shows considerable variation in quality, and most videos provide incomplete or inaccurate guidance regarding diagnosis and management. Patients relying on these videos may encounter persuasive narratives that normalize symptoms or promote ineffective or harmful management strategies rather than encouraging cannabis cessation and medical evaluation. Clinicians should anticipate that patients have likely been exposed to such content, directly address misconceptions, and guide them toward vetted educational resources. High-quality, expert-developed CHS content is needed to improve the reliability of information available on social media platforms.

## Introduction

Cannabis use continues to increase as medical and recreational legalization expands both in the United States and globally [1-3]. Although cannabis is used for several therapeutic indications, adverse effects have also become more apparent, including cannabinoid hyperemesis syndrome (CHS). CHS is characterized by cyclic vomiting in people with chronic, heavy cannabis use and is often accompanied by compulsive hot bathing for symptom relief [4-6]. The condition can lead to repeated emergency department visits, extensive diagnostic testing, and substantial healthcare costs, yet it remains underrecognized and inconsistently diagnosed [6,7]. Delayed recognition may delay counseling on cannabis cessation, the most reliable treatment for CHS.

YouTube (Alphabet Inc., Mountain View, CA) has become a major source of health information, and health-related videos increasingly influence patient understanding and decision-making [8-13]. Recent survey data indicate that approximately 87.6% of YouTube users report watching health-related content, and 84.7% state that these videos affect their health-related decisions, including whether to consult a clinician or change health behaviors [9,10]. At the same time, prior studies have shown that medical content on YouTube is often incomplete, biased, or inaccurate, particularly when produced by nonprofessional or commercial sources [14-17]. Cannabis-related videos are especially prominent, often attracting high engagement while providing limited discussion of risks or containing misinformation [8,18]. Prior work has also identified CHS-related misinformation online, including unsupported explanations for disease causation, minimization of symptom severity, and promotion of ineffective or potentially harmful self-management strategies [18-19]. For someone desperately searching for an explanation for their symptoms, these videos can be persuasive, even when incomplete or inaccurate.

This issue is clinically important because patients with suspected CHS may turn to online videos to interpret symptoms, consider treatment options, and decide whether to seek medical care [18]. Given that CHS may be unfamiliar to some clinicians and challenging for some patients to accept, online content may meaningfully shape risk perception and the willingness to cease cannabis use [13,18]. However, despite the growing volume of CHS-related videos, their accuracy, completeness, and clinical usefulness remain poorly characterized [19,20].

The objective of this study was to evaluate the accuracy, completeness, and clinical usefulness of YouTube videos about CHS. Specifically, the analysis assessed whether videos provided accurate information about diagnosis, treatment, prevention, complications, and indications for seeking medical care, and whether they contained misleading or unsubstantiated medical claims.

## Materials and methods

Study design

This cross-sectional content analysis evaluated CHS-related videos on YouTube. The Michigan State University Institutional Review Board reviewed the study protocol and determined that it constituted non-human subjects research.

Search strategy

YouTube searches were conducted using 15 predefined CHS-related search prompts, including both clinical and lay terms (Table 1). The platform’s search function ranks videos based on a combination of keyword relevance, engagement metrics, and user-specific factors, such as watch history and location. This algorithm makes it difficult to obtain a completely unbiased or exhaustive list of videos on a given topic. To minimize personalization, all searches were conducted in a logged-out browser with a cleared cache and default settings. For each term, the first 50 relevance-ranked results were screened using protocol-specified inclusion and exclusion criteria. Screening was stopped when additional scrolling yielded only duplicates or clearly irrelevant content. This approach aligns with published methods for YouTube health content analyses and systematic gray literature searching [18,21-23].

**Table 1:** YouTube search queries for CHS-related content CHS: cannabinoid hyperemesis syndrome

Query search terms
Cannabinoid hyperemesis syndrome
Cannabis hyperemesis syndrome
Marijuana hyperemesis syndrome
Cannabinoid hyperemesis
CHS vomiting
CHS cannabis
CHS weed
Vomiting from weed
Throwing up from weed
Weed makes me vomit
Cannabis nausea and vomiting
Cyclic vomiting from cannabis
Can’t stop throwing up after smoking weed
Hot showers stop vomiting weed
How to stop CHS

Video content

Videos were collected from April to September 2023. Extracted variables included views, content author or publisher, presenter format, likes, dislikes, comments, and abstractor impressions. For each video, the total number of viewer comments was recorded and used as a quantitative indicator of engagement. Medical claims were classified as either substantiated or unsubstantiated by two board-certified toxicologists (BSJ, BDR) with extensive experience in cannabis-related toxicology.

Training of content reviewers

All investigators were trained in their protocol roles and jointly reviewed the abstraction form, coding scheme, and eligibility criteria at study initiation. Student reviewers completed protocol-specific training covering data extraction, use of the accuracy checklist, and content analysis principles. To standardize baseline knowledge and support consistent identification of CHS-related content, reviewers received a brief CHS fact sheet summarizing diagnostic features, clinical course, common misconceptions, and key management points. Final judgments about accuracy and substantiation were made by the toxicology experts.

Training included a piloting phase in which student reviewers independently coded a small set of videos, with discrepancies resolved through group discussions with senior investigators. Based on this iterative feedback, wording and operational definitions were refined before full data collection began. Throughout the abstraction period, seven student reviewers were mentored by a senior author, and a faculty member periodically reviewed forms to answer questions, monitor quality, and minimize interobserver variation.

Data abstraction

A standardized .docx-based abstraction form was organized into four sections. The first captured video characteristics, including URL, date accessed, date published, source, length, views, comments, likes, dislikes (when available), presentation format, and presenter demographics. The second section assessed content accuracy using a 16-item, open-ended response CHS checklist covering definition, epidemiology, symptoms, diagnosis, treatment, prevention, complications, and indications for seeking care (Table 2). Videos were classified based on the number of criteria met: ≥13 items as exemplary, 10-12 as useful, 7-9 as not useful, and ≤6 or any video containing unsubstantiated claims as misleading. These thresholds were developed a priori, informed by frameworks such as DISCERN and the Global Quality Scale [24-27], and were reviewed and approved by two medical toxicologists to ensure expert content validity.

**Table 2:** Topic accuracy assessment CHS: cannabinoid hyperemesis syndrome; ER: emergency room

Topic accuracy assessment
What is cannabis hyperemesis syndrome?
Who might get cannabis hyperemesis syndrome?
How common is cannabinoid hyperemesis syndrome?
What causes cannabis hyperemesis syndrome?
What are the symptoms of CHS?
How is cannabis hyperemesis syndrome diagnosed?
Is there a cannabis hyperemesis syndrome cure?
How is CHS treated?
Can I treat CHS symptoms at home?
How soon after cannabis hyperemesis syndrome treatment will I feel better?
How can I prevent cannabis hyperemesis syndrome?
What are the possible complications of CHS?
When should I go to the ER?
Tips for dealing with CHS symptoms
Referral to an addiction clinic or facility
Warning that severe cannabis hyperemesis syndrome can be fatal?

The third section captured free-text responses documenting inaccurate or misleading information as well as helpful or unhelpful aspects. Each video received an overall usefulness rating (useful, not useful, misleading) as outlined in Table 3. All assessment tools were developed by the authors specifically for this study, informed by prior work evaluating online medical content, and have not been formally validated as independent instruments [28-30].

**Table 3:** Clinical usefulness rating scale

How would you rate this video?	Rating
Additional instructions: Please rate this video as useful if it provides accurate and helpful information about symptoms, risks, or treatments; not useful if it discusses CHS but lacks clinical relevance; or misleading if it contains false information	
▪ Evidence-based and/or informative results for symptomatology, risks, and treatment	Useful
▪ Results regarding CHS, but not clinically relevant	Not useful
▪ False information	Misleading

Reviewers often described a video as biased or misleading when it strongly promoted a particular viewpoint, minimized risks, or appeared to serve primarily as an advertisement, regardless of production quality or narrative style. Student impressions of bias were informed by their CHS training and fact sheet, but final classification of specific statements as unsubstantiated or misleading was determined by the toxicologist.

Following abstraction piloting, videos were assigned to student reviewers for parallel abstraction. Disagreements were discussed in review meetings and resolved by consensus. No conflicts required mediation by an additional author. Responses were then entered into a spreadsheet for thematic coding and quantitative analysis. To assess interrater reliability, 10% of videos were independently reviewed by senior physician investigators, and Cohen's kappa was calculated to quantify agreement beyond chance [31].

Medical claim substantiation

To assess the veracity of medical statements in the video content, two board-certified toxicologists (BSJ, BDR) independently assessed medical statements. A statement was considered substantiated if clearly supported by rigorous, up-to-date scientific evidence and aligned with expert consensus [30,32,33]. Review criteria for unsubstantiated claims are shown in Table 4.

**Table 4:** Review criteria for unsubstantiated claims

Review criteria for unsubstantiated medical claims	
Definition	Description
Absence of evidence	The claim is not supported by systematic reviews, randomized controlled trials, well-designed observational studies, or official statements from recognizable health authorities
Contradicted by scientific consensus	The statement is inconsistent with or directly contradicts the current consensus in the relevant medical or scientific community
Reliance on anecdote or low-quality sources	The claim is based solely on anecdotal reports, non-peer-reviewed sources, outdated information, or expert opinion without corroborating evidence
Lack of reproducibility	The statement cannot be consistently verified across multiple, independent, and high-quality sources
Ambiguity of exaggeration	The claim is vague, misleading, or exaggerated beyond what available evidence supports

Study endpoint and analysis

The primary endpoint was overall clinical utility, defined as how clearly and accurately videos described CHS, including associated risks and complications. The main outcome was the reviewer-rated quality and educational value of each video for clinical information about CHS, as assessed by our trained reviewers and expert toxicologists. Descriptive statistics summarized key variables with 95% confidence intervals (CIs). Associations between continuous variables were examined using Pearson product-moment correlation coefficients.

## Results

YouTube content characteristics

Over 900 potentially eligible videos were identified in a pre-study search. Of these, 100 videos initially met the inclusion criteria. Three were excluded (one duplicate, one set to private, one irrelevant), yielding a final sample of 97 videos. The videos were collectively viewed a total of 795,264 times, with an average of 8,284 and a 1,050 median (interquartile range (IQR) of 8,284 (254-8,304) views noted per video. The mean video length was 9.8 ± 4.1 minutes (0.60-47.7 minutes). These video metrics fell within the site averages for all content uploaded to the platform [34,35].

The average age of the content (time from upload to access) was 30 ± 2.1 months (range: <1-85 months). Over half of the videos (n = 50, 52%) were accessed within two years of publication. Content “likes” had a mean of 257 (range: 0-8,400) and a median (IQR) of 28 (7-218) per video, with three videos having unavailable counts and two videos where uploaders had disabled counts. Dislike counts were available for only 26 videos uploaded before November 2021, when YouTube disabled dislike counts (mean: 1, range: 0-11).

Most videos (n = 82, 85%) featured on-camera presenters (character format), with 70 (72%) narrated by Caucasian presenters. Other demographic groups made up approximately 27 (28%) of the character videos. Non-character presentations (n = 15, 15%) included animations (n = 19, 20%) and photographs or slideshows (n = 3, 3%).

Engagement metrics and video quality

To assess the presence of relationships between content metrics, Pearson product-moment correlation coefficient (PPMCC) testing was performed. This analysis (Table 5) revealed no meaningful linear relationships between content age, video length, views, comments, or reviewer-rated usefulness (all correlations near 0).

**Table 5:** PPMCC for content metrics PPMCC: Pearson product-moment correlation coefficient; CI: confidence interval

Comparison	PPMCC (95% CI) (r)	Lower bound (a = 2.5%)	Upper bound (a = 2.5%)
Content age (months) vs. number of views	0.1384	-0.062	0.328
Length (minutes) vs. number of views	0.0797	-0.121	0.274
Length (minutes) vs. number of comments	0.1651	-0.035	0.352
Length (minutes) vs. rating	0.0697	-0.131	0.265
Number of views vs. rating	-0.2082	-0.391	-0.01

Accuracy and completeness

No video met all 16 checklist criteria. Only two videos (2.1%) were classified as exemplary (≥13 accurate items), 25 (25.8%) as useful (10-12 items), 51 (52.6%) as not useful (seven to nine items), and 19 (19.6%) as misleading (≤6 items or containing unsubstantiated claims). Table 6 shows the coverage of individual checklist items; the most frequently addressed topics were hospital treatment (78%), definition (66%), and cure information (65%), while the least covered were fatal warnings (6%), addiction referrals (9%), and indications for emergency care (11%).

**Table 6:** Accurate information checklist: number of videos and percentage of covered content ^*^Percentages indicate the fraction of videos that mentioned the topic and did so with content judged to be accurate by reviewers CHS: cannabis hyperemesis syndrome

Accurate information checklist	N	%^*^
How is cannabis hyperemesis syndrome treated in the hospital?	76	78%
What is cannabis hyperemesis syndrome?	64	66%
Is there a cure for cannabis hyperemesis syndrome?	63	65%
Who might get cannabis hyperemesis syndrome?	51	53%
What causes cannabis hyperemesis syndrome?	50	52%
What are the symptoms of cannabis hyperemesis syndrome?	48	49%
How can someone prevent cannabis hyperemesis syndrome?	37	38%
Tips for dealing with cannabis hyperemesis syndrome symptoms?	32	33%
Treating cannabis hyperemesis syndrome symptoms at home?	31	32%
How soon after treatment for CHS will someone feel better?	26	27%
What are the possible complications of cannabis hyperemesis syndrome?	23	24%
How is cannabis hyperemesis syndrome diagnosed?	20	21%
How common is cannabis hyperemesis syndrome?	17	17%
When should someone go to the emergency department for CHS?	11	11%
Referral to an addiction clinic or facility?	9	9%
Warning that severe cannabis hyperemesis syndrome can be fatal?	6	6%

Video sources and formats

The study characterized the sources and perspectives of the videos, providing clinicians with context useful for discussing CHS with patients. Testimonials comprised 79% of videos (n = 77), primarily from current or former patients (n = 58, 60%). These candid discussions were often unstructured and included opinions and descriptions of clinical encounters. Ten testimonials functioned as advertisements for a for-profit substance-use coaching program. Didactic videos (n = 15, 15%) included six local news briefs that provided concise, useful overviews. Eight videos featured healthcare professionals (five physicians, one nurse, one pharmacist, and one EMT), although professional credentials did not guarantee the accuracy of the information presented.

Clinical usefulness, medical substantiation, and misleading claims

Strict definitions were applied to determine clinical usefulness. Content was required to satisfy at least 75% of the checklist (see topic accuracy assessment Table above) and meet standards for medical substantiation. Overall, 26% (n = 25) of videos were rated useful, 54% (n = 52) not useful, and 20% (n = 20) were considered misleading. Inaccurate information appeared in 55% of videos (n = 53). Interrater agreement was substantial for usefulness classification (κ = 0.78, 95% CI: 0.66-0.90) and for the presence of inaccurate information (κ = 0.71, 95% CI: 0.61-0.82).

Of the 58 patient testimonials, 15 (26%) contained unsubstantiated claims about CHS etiology or management, including denial of CHS existence, attribution to contaminated cannabis, claims that hot showers or home remedies permanently cure CHS, assertions that only heavy users are at risk, or statements that any vomiting in cannabis users indicates CHS.

Thematic analysis of reviewer impressions

An impartial qualitative assessment of the free-text responses was conducted by a faculty non-reviewer. The following themes and subcategories were identified for key reviewer questions. Reviewers identified both positive and problematic content features (Tables 7-8). Favorable elements included personal anecdotes that conveyed relatability and honesty about struggles with addiction (n = 95, 98% had some favorable features), accurate medical information, and high production quality with concise format (that is, less than <5 minutes in duration). Unfavorable aspects included off-topic or overly informal presentation, coarse language, poor audiovisual quality, and biased or misleading claims, particularly in videos promoting products or services. For 12 reviewers, 12% "no negative" impressions were documented.

**Table 7:** Selected impressions from thematic analysis: what did you like about the video? CHS: cannabis hyperemesis syndrome

Identified theme	Selected impressions
Personal anecdotes: relatability	“I think it’s always nice to hear from patients about their experience and what’s helped them because that can give us insight into things we might be missing… as clinicians.”
	“Honest discussion on the psychological toll CHS takes, including depression and self-harm.”
Accurate information	“informational, accurate”
	"Clearly and succinctly explained (most of) the major points of CHS.”
Format (production value)	“Short and informative, useful for a two-minute overview of CHS.”
	“Professionally produced, presented clearly and succinctly.”

**Table 8:** Selected impressions from thematic analysis: what did you dislike about the video?

Identified theme	Selected impressions
Off-topic/too informal	“Poor cinematic quality”
	“Vulgarity may make the video unprofessional in some circumstances.”
Bias and misleading claims	“Overtly biased toward seeking positive effects of marijuana while discounting its risks and negative effects.”
	“Very much a sales video for their addiction recovery services.”

Inaccurate information (n = 55, 57%) most commonly involved unsubstantiated etiologies (pesticides, genetic changes, tetrahydrocannabinol (THC) release theories), unproven claims about cannabidiol (CBD) products, downplaying symptom severity, recommendations for continued cannabis use (including one advocating increased intake), and suggestions that lifestyle or diet changes alone are sufficient treatment instead of complete cessation. These impressions are outlined in Table 9 below.

**Table 9:** Selected impressions from thematic analysis: any inaccurate information? CHS: cannabis hyperemesis syndrome

Identified theme	Selected impressions
Causes and symptoms	“States that CHS does not exist, and the symptoms are due to stomach ulcers due to smoke intake.”
	“Says you can stop CHS by stopping smoking for a month and then lowering intake afterwards.”
Treatment	”Basically encouraged continued use, saying that the home remedies will make it better enough to not need to stop smoking.”
	“Says CHS can heal in different ways and wants to believe there is a way to cure CHS without stopping the use of marijuana.”

## Discussion

CHS is increasingly recognized as a consequence of chronic, heavy cannabis use, yet timely diagnosis and acceptance of cannabis cessation as the primary treatment remain challenging. Patients with suspected CHS commonly search online for explanations of their symptoms before or between clinical encounters, often turning to YouTube for first-person accounts and advice. This study provides, to our knowledge, the first systematic evaluation of CHS-related YouTube videos. The findings reveal substantial gaps in reliable information, a high prevalence of misleading claims, and weak correlations between video popularity and medical quality.

Main findings

Of 97 CHS-related YouTube videos analyzed, only a small minority were clinically useful or exemplary; most were rated not useful or misleading. Videos frequently omitted essential information, including explicit diagnostic criteria, indications for urgent evaluation, evidence-based management strategies, and the central role of sustained cannabis cessation. Even when videos accurately described some CHS features, they often lacked critical content domains, limiting their value as patient education resources.

Personal testimonial videos were common and often valued by reviewers for their honesty and relatability, conveying the emotional toll of recurrent vomiting, emergency visits, and stigma associated with cannabis use. However, testimonials also accounted for a substantial proportion of unsubstantiated claims, including speculative etiologies, minimized symptom severity, and promotion of unproven remedies. This duality illustrates how patient narratives can both validate lived experiences and amplify misinformation about CHS [32].

Standard YouTube engagement metrics (views, likes, comments) showed no meaningful linear association with reviewer-rated usefulness. Several highly viewed videos contained substantial misinformation, suggesting that basic engagement metrics are unreliable proxies for content quality. This disconnect underscores the challenge patients face when using surface cues to judge credibility.

Clinical and educational implications

Given the high rates of online health information seeking, clinicians should assume that many patients have viewed CHS-related videos and directly ask about content exposure to clarify misconceptions, align expectations, and build trust. Common themes requiring discussion include attributing CHS to contaminated cannabis rather than chronic use, overemphasizing hot showers or home remedies as curative, and suggesting symptom control without cannabis cessation.

Because engagement metrics do not reliably indicate content quality, clinicians should recommend specific, vetted resources rather than expecting patients to identify high-quality content independently [28,30]. Professional societies and educators have an opportunity to address this gap by creating concise, evidence-based, patient-facing content that is easy to access. Such resources should detail diagnostic features, risks, management rationale, and indications for urgent care, and ideally be readily available on the same content platforms [27]. Collaboration between toxicologists, emergency physicians, and communications professionals could also engage active patients to discuss their experiences with seeking health information, which may enhance both the accuracy of CHS patients’ lived experiences and the content’s audience appeal.

Strengths and limitations

This study employed a transparent, systematically applied search strategy with a structured CHS content checklist informed by expert toxicologists and supported by standardized reviewer training. It also demonstrated substantial interrater agreement (κ = 0.78). Additionally, the mixed-methods approach to reviewer ratings and impressions provided a nuanced understanding of how CHS is portrayed on YouTube.

At the same time, several limitations should be considered when interpreting these findings. The author-developed checklist and usefulness scale, while supported by expert input and high kappa values, were study-specific and have not undergone formal psychometric validation. Classification thresholds were set a priori and deliberately conservative, and different cutoffs could alter the distribution of categories.

YouTube search results are dynamic and influenced by personalization factors that cannot be fully controlled. Despite using standardized search terms and logged-out browsing, the dataset represents a time-limited snapshot (April-September 2023) and may reflect selection and platform algorithm biases. The analysis was restricted to English-language content on a single platform, limiting generalizability to other languages, regions, or platforms such as TikTok or Instagram. The modest sample size (n = 97) is adequate for descriptive analysis but constrains statistical power for detecting small associations or subgroup analyses. Our analyses are therefore primarily descriptive, supplemented by simple correlation tests, and are not intended to support complex inferential modeling.

Additionally, there were a few YouTube policy changes that affected the content included in this analysis. YouTube disabled the “dislike” reaction in November 2021, which affected 27 videos (26%) of our dataset. Further, a Community Guidelines update in August 2023, during the data extraction period, provided a framework for prevention, treatment, and disinformation categorization for reporting violations [36]. While our dataset was not actively affected by these changes, future content analyses will likely be influenced by these updates.

Judgments of usefulness and misleading content involve some degree of subjectivity despite structured tools, reviewer training, and consensus procedures. This may contribute to misclassification at the margins and should be considered when interpreting the exact proportions of videos in each category. Finally, the study characterizes content but does not assess how viewers interpret or act on videos, precluding causal inferences about clinical outcomes. Our recommendations to clinicians and educators should therefore be understood as reasoned implications of the observed content landscape rather than conclusions about measured behavioral effects.

Future research directions

Future research could analyze larger, more diverse samples, including non-English content and videos on other platforms; examine trends over time to determine whether content quality improves in response to platform policy changes or professional education initiatives; and formally validate assessment tools against established measures, such as DISCERN, to enhance comparability across studies.

## Conclusions

Most CHS-related YouTube videos in this sample were not clinically useful and frequently contained incomplete or unsubstantiated information regarding diagnosis, risks, and management. Personal testimonials often blend accurate symptom descriptions with speculative etiologies and non-evidence-based advice. Because engagement metrics are poor indicators of content quality, patients relying on popular videos may encounter misleading messages that downplay the importance of cannabis cessation and medical evaluation. Clinicians should anticipate patient exposure to such content, address misconceptions directly, and guide patients toward vetted resources. High-quality, expert-developed CHS educational videos are urgently needed to improve the reliability of information available on social media platforms.
